# Supplementary material for: Identification of Repurposable Drugs and Adverse Drug Reactions for Various Courses of COVID-19 Based on Single-Cell RNA Sequencing Data
Source: ArXiv. 2020 May 16:arXiv:2005.07856v2. Preprint. [Version 2] (PMC7724679)

SUPPLEMENTARY INFORMATION

Supplementary Figure S1. UMAP presentation of a single-cell atlas of BALFs showing 6 major cell types.

Supplementary Figure S2. The violin plot shows the expression of signature genes (CD68, IL7R, CD4, CD8A, MS4A1, and TPPP3) from major cell types.

Supplementary Figure S3. The antiviral activities of tyrphostin-AG-1478 and brefeldin-a against IAV in vitro. (A-C) Antiviral activity and dose-response curves of tyrphostin-AG-1478 (AG-1478, 0 to 10 μM) and brefeldin-a (BFA, 0 to 10 μM) in Calu-3 cells (B) and Vero-E6 cells (A, C) infected with IAV PR8 (H1N1, MOI of 1) at 24 hpi. Cell viability is depicted in black. The IC_{50} was calculated based on normalization to the control and fitted in GraphPad Prism. (D) Bar plots showing mRNA levels of cellular *ACE2*, *MX1*, *ISG15*, and *IFNB1,* calculated with ΔΔCT over noninfected Calu-3 cells. Calu-3 cells were treated with BFA (0 to 10 μM) and infected with IAV (MOI of 1). Data are represented as the mean ± SD with 3 technical replicates each. Statistical significance was determined using one-way ANOVA. $^{**}P < 0.01$, $^{****}P < 0.0001$.

Supplemental Table S1. Clinical data of the enrolled subjects (SARS-COV-2 confirmed).

Supplemental Table S2. The numbers of differentially expressed genes in different cell types (clusters) among three group comparisons.

Supplemental Table S3. Differentially expressed genes between mild and healthy samples in B cells, $CD4^+$ T cells, $CD8^+$ T cells, epithelial cells, NK cells and macrophages.

Supplemental Table S4. Differentially expressed genes between severe and healthy samples in B cells, $CD4^+$ T cells, $CD8^+$ T cells, epithelial cells, NK cells and macrophages.

Supplemental Table S5. Differentially expressed genes between severe and mild samples in B cells, $CD4^+$ T cells, $CD8^+$ T cells, epithelial cells, NK cells and macrophages.

Supplemental Table S6. Full list of overlapping potential drugs among three comparisons (mild vs healthy, severe vs healthy and severe vs mild).

Supplemental Table S7. Full list of potential drugs for treating COVID-19 based on the LINCS database and DEGs between mild and healthy samples in B cells, $CD4^+$ T cells, $CD8^+$ T cells, epithelial cells, NK cells and macrophages. Connectivity scores were calculated from the CLUE platform.

Supplemental Table S8. Overlapping potential drugs based on DEGs among different cell clusters between mild and healthy patients.

Supplemental Table S9. Full list of potential drugs for treating COVID-19 based on the LINCS database and DEGs between severe and healthy samples in B cells, $CD4^+$ T cells, $CD8^+$ T cells, epithelial cells, NK cells and macrophages. Connectivity scores were calculated from the CLUE platform.

Supplemental Table S10. Overlapping potential drugs based on DEGs among different cell clusters between severe and healthy patients.

Supplemental Table S11. Full list of potential drugs for treating COVID-19 based on the LINCS database and DEGs between severe and mild samples in B cells, $CD4^+$ T cells, $CD8^+$ T cells, epithelial cells, NK cells and macrophages. Connectivity scores were calculated from the CLUE platform.

Supplemental Table S12. Overlapping potential drugs based on DEGs among different cell clusters between severe and mild patients.

Supplemental Table S13. A list of potential drugs for treating COVID-19 based on DEGs from RNA-seq data between patient and healthy samples and the LINCS database.

Supplemental Table S14. A complete list of on-label ADRs of all candidates. On-label ADRs of drugs were downloaded from the Side Effect Resource (SIDER). The ADR terms are mapped to Preferred Terms (PTs) coded in MedDRA v16.0.

Supplemental Table S15. A complete list of off-label ADRs of all candidates. Off-label ADRs were processed data from the postmarket ADR reports within the FDA Adverse Event Report System (FAERS). The ADR terms are mapped to Preferred Terms (PTs) coded in MedDRA v16.0.

Supplementary Figure S1

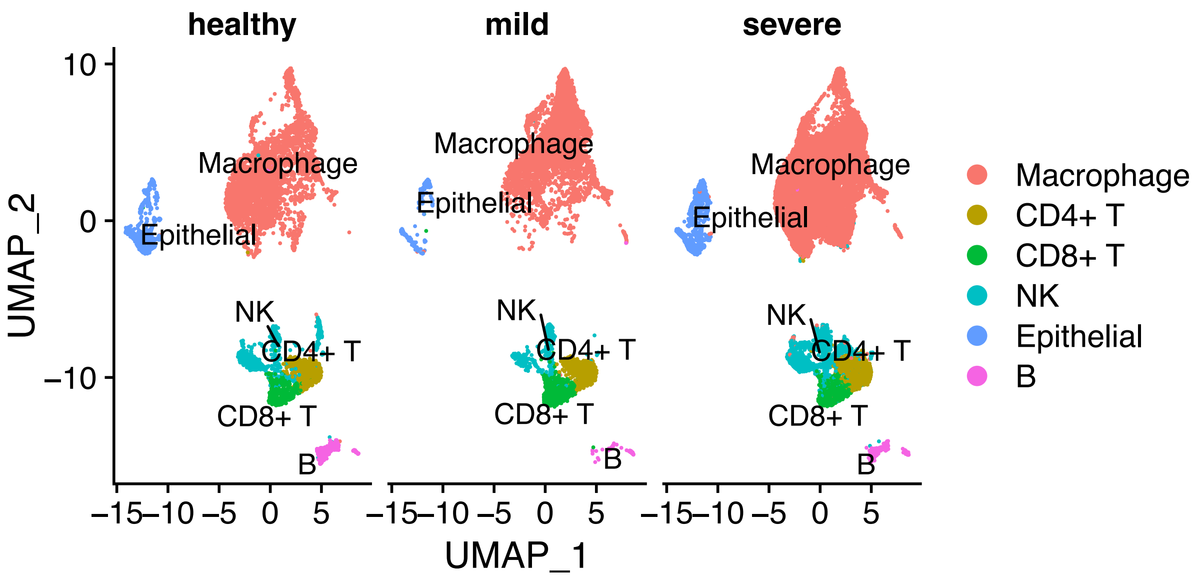

Supplementary Figure S2

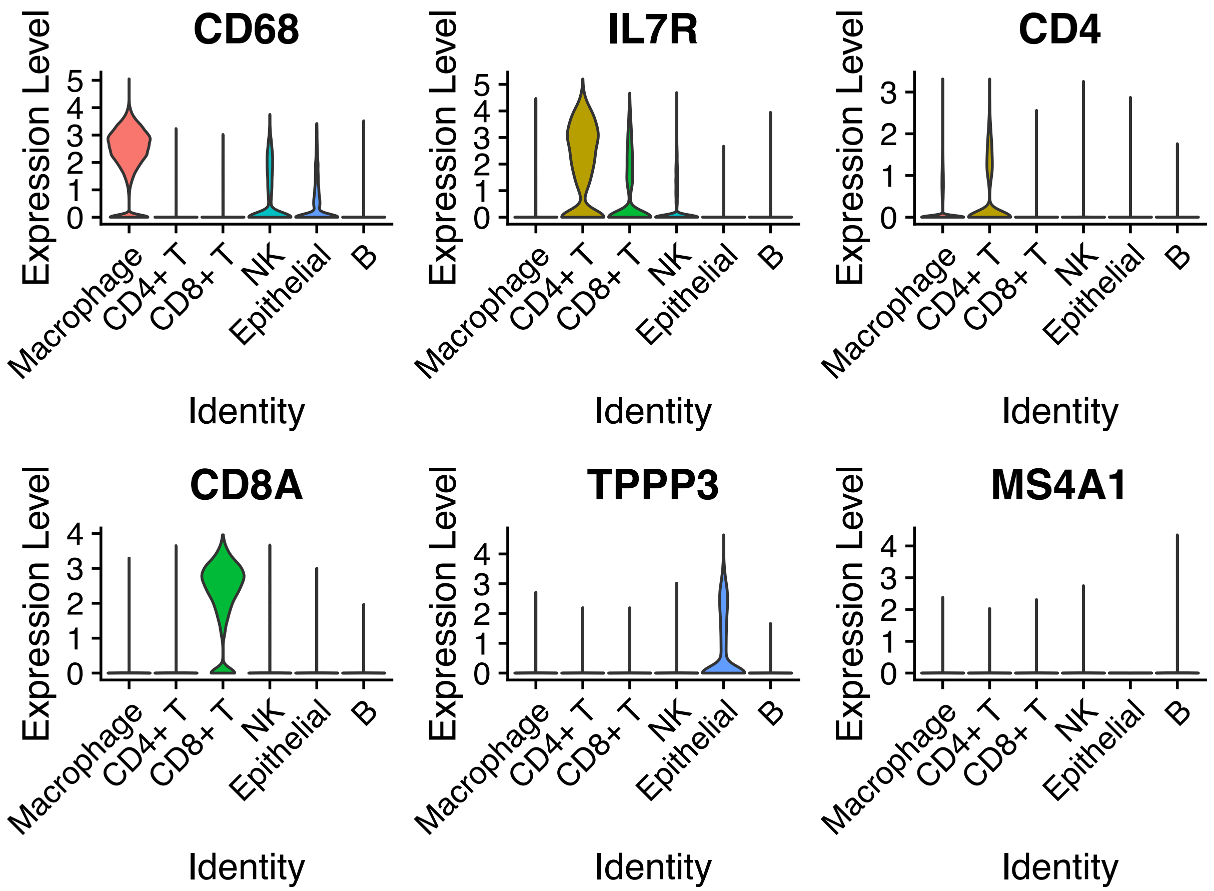

Supplementary Figure S3

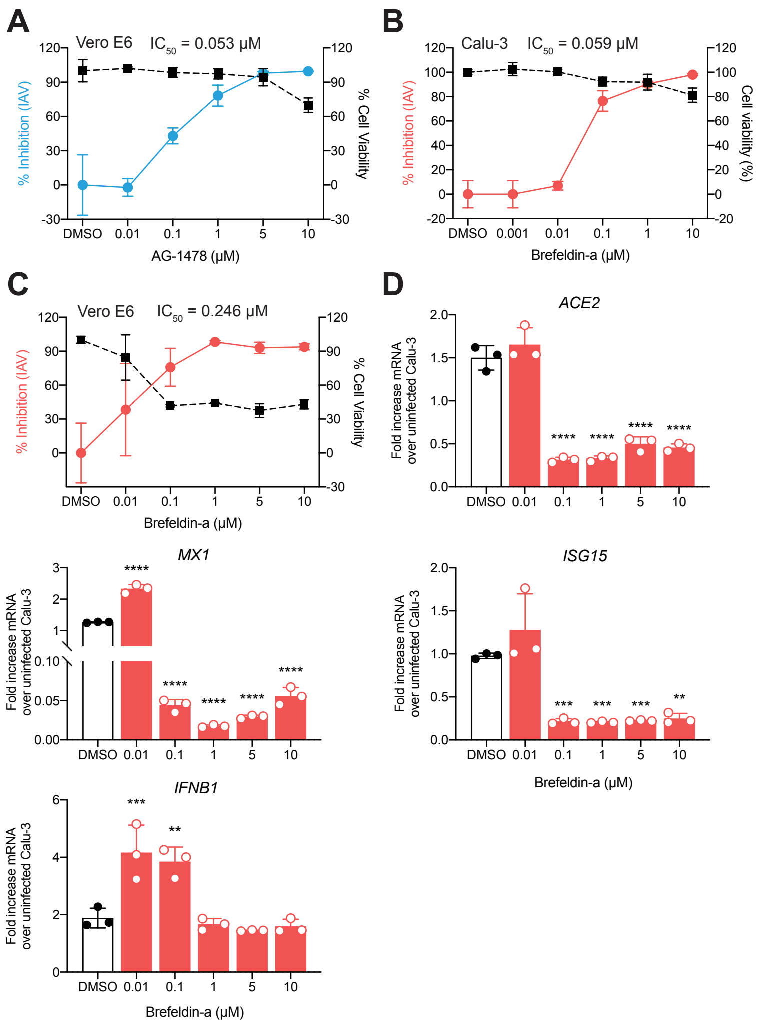

Supplement: 1 [file NIHPP2005.07856V2-supplement-1.pdf]
